# Vibration, current, torque, RPM dataset for multiple fault conditions in industrial-scale electric motors under randomized speed and load variations

**DOI:** 10.1016/j.dib.2025.111954

**Published:** 2025-08-06

**Authors:** Wonho Jung, Junho Kim, Kangmin Jang, Sung-Hyun Yun, Daeguen Lim, Minje Jin, Yong-Hwa Park

**Affiliations:** a Center for Noise and Vibration Control Plus, Department of Mechanical Engineering, Korea Advanced Institute of Science and Technology, 291, Daehak-ro, Yuseong-gu, Daejeon 34141, South Korea; b Digital Appliances, Samsung Electronics, 129, Samsung-ro, Yeongtong-gu, Suwon-si, Gyeonggi-do 16677, South Korea

**Keywords:** AC motor, HVAC, Load fluctuation, Variable frequency drive, Condition monitoring

## Abstract

This article presents a comprehensive dataset acquired from two fault diagnosis environments: (1) industrial AC motors operating under various real-world conditions, and (2) belt-loosening scenarios in HVAC air handling units. The dataset was collected to support the development and validation of data-driven fault detection methods across diverse mechanical and electrical systems. For the AC motor dataset, faults were deliberately introduced to simulate common degradation modes, including coil winding faults, inter-phase short circuits, misalignment, and bearing-related issues such as rolling-element and journal bearing faults. Each fault was replicated with multiple severity levels. Data were collected under randomized speed fluctuations (6 % and 16 %) using variable frequency drive. Data were also collected under variable load conditions, and different motor capacity. The recorded sensor signals include three-phase current data (R-, S-, T-phase), vibration data (z-axis), and torque data. The HVAC dataset focuses on belt-loosening faults within air handling units and includes vibration data (x-, y-axis), current data (R-, S-, T-phase), and RPM (motor part, fan part) under varying belt tension levels. The dataset comprises over 60 GB of raw signals with current sampled at 100 kHz, vibration and torque at 25.6 kHz, and RPM at 100 kHz. Each test scenario ranges from 120 to 300 s, resulting in various of labeled data segments suitable for training and benchmarking machine learning models. Unlike existing public datasets that often assume constant speed or isolated fault types, this dataset uniquely incorporates multi-fault, multi-severity conditions under randomized speed/load variations, filling critical gaps in real-world applicability for robust fault diagnosis algorithms. The dataset enables robust evaluation of machine learning models and signal processing algorithms for fault detection, condition monitoring, and predictive maintenance in rotating machinery. The inclusion of multi-fault, multi-severity, and variable-condition data makes it especially suitable for training generalizable diagnostic algorithms in both academic and industrial contexts. Metadata and labeling for fault type, severity, and operating conditions are provided to facilitate supervised learning applications.

Specifications Table

**Table:** 

Subject	Engineering & Materials science
Specific subject area	*Rotating Machine Condition Monitoring*
Type of data	Table, Image, Chart, Graph, Figure, Raw
Data collection	This testbed can emulate winding faults, bearing faults, shaft parallel misalignment faults, journal bearing faults, and belt-loosening faults. The dataset is composed of two parts. First dataset was acquired from the testbed under different load and constant rotating speed condition. Second dataset was acquired from the testbed under different load and under randomly varying rotating speed condition.
Data source location	Institution: Human Lab., Center for Noise and Vibration Control Plus, Department of Mechanical Engineering, Korea Advanced Institute of Science and Technology (KAIST) · City: Daejeon · Country: South Korea
Data accessibility	Repository name: AC Motor Testbed under constant or varying speed (AC.zip, AC.z01, AC.z02, AC.z03, AC.z04, AC.z05, AC.z06) Data identification number: 10.17632/9r82jppsn7.1, 10.17632/wg6h28cfbs.1, 10.17632/mvdg4t6cz7.1, 10.17632/697w94bhw2.1, 10.17632/j2r275rmdf.1, 10.17632/gr8c9ckhxh.1, 10.17632/j8c6mf2gn8.1, Direct URL to data: https://data.mendeley.com/datasets/9r82jppsn7/1 https://data.mendeley.com/datasets/wg6h28cfbs/1 https://data.mendeley.com/datasets/mvdg4t6cz7/1 https://data.mendeley.com/datasets/697w94bhw2/1 https://data.mendeley.com/datasets/j2r275rmdf/1 https://data.mendeley.com/datasets/gr8c9ckhxh/1 https://data.mendeley.com/datasets/j8c6mf2gn8/1 Repository name: Belt Looseness Fault Simulation in an HVAC Testbed Data identification number: 10.17632/5tt3zb2pns.1 Direct URL to data: https://data.mendeley.com/datasets/5tt3zb2pns/1
Related research article	Sung Hyun Yun, Wonho Jung, Daeguen Lim, and Yong-Hwa Park, “Anomaly Detection for Belt Looseness Using Motor Current Signal Imaging in Air Handling Unit,” in *Proceedings of the 4th Asia Pacific Conference of the Prognostics and Health Management*, Tokyo, Japan, September 2023.

## 1 Value of the Data

• Comprehensive fault simulation under real-world operating conditions: This dataset includes electric motor and HVAC system faults collected under realistic variations. Unlike many datasets that assume constant operating environments, these data replicate dynamic industrial conditions, allowing researchers to develop and validate more robust fault diagnosis models that generalize to real-world use cases.
• Multi-modal sensor signals for advanced diagnostic algorithm development: The dataset comprises synchronized measurements of three-phase currents (R-, S-, T-phase), vibration signals (x-, y-axis), RPM, and torque. This rich sensor fusion allows researchers to explore cross-domain signal relationships and design multi-input diagnostic frameworks, such as deep learning models or hybrid physics-informed AI approaches.
• Diverse fault types and graded severity levels: The data include critical fault types such as coil winding faults, inter-turn shorts, bearing damage (including rolling-element and journal bearings), misalignment, and HVAC belt loosening. Each fault is recorded at multiple severity levels, enabling benchmarking of fault classification models under progressive degradation scenarios.
• Suitable for transfer learning and domain adaptation studies: Because the dataset spans two different domains—AC motors and HVAC systems—researchers in the field of transfer learning, domain adaptation, and generalized fault detection can leverage the structural similarities and domain discrepancies to test algorithm adaptability across mechanical systems.
• Label-rich dataset ready for supervised learning applications: Each data segment is annotated with detailed metadata, including fault type, severity grade, motor/load configuration, and operational conditions. This facilitates straightforward use in supervised learning tasks, algorithm benchmarking, and reproducibility studies in fault diagnosis research.
• This dataset could be particularly valuable to researchers in the field of electric motor fault diagnosis, including those working in predictive maintenance for HVAC systems. Industrial stakeholders may benefit from applying this data to develop early fault detection algorithms. Furthermore, academic institutions with research groups in signal processing and mechanical engineering can utilize this dataset for machine learning model development and benchmarking.

## 2 Background

The compilation of this dataset was motivated by a comprehensive review of fault prevalence in electric motors as reported in industrial and academic sources over the past several decades. According to multiple large-scale surveys—including OEM FMEA analyses, MOD and IEEE motor studies, and utility application reviews—bearing-related faults consistently represent the most common failure mode across motor sizes and applications, followed by electrical insulation faults and rotor-related issues. However, the analysis of 80 IEEE and IEE journal papers published over the past 26 years reveals a research bias favoring stator and insulation failures, despite the higher industrial occurrence of mechanical faults such as bearing wear and rotor imbalance as shown in Table 1.

**Table 1:** Failure rate by motor part.

Parts	Predicted by an OEM through FMEA techniques, 1995–7 [1]	MOD survey, tavner, 1999 [2]	IEEE large motor survey, 1985, O’Donnell, 1985 [3]	Motors in Utility Applications, Albrecht, 1986 [4]	Motor Survey Offshore and Petrochemical, Thorsen, 1995 [5]\	Proportion of 80 Journal Papers published in IEEE and IEE on these subject areas over the past 26 years
Motor types	Small LV Motors & Generators, ≤150 kW, Agricultural & Industrial LV Induction Motors	Medium LV Motors & Generators, ≤750 kW, Agricultural & Industrial LV Induction Motors	≥150 kW Motors, MV & HV Induction Motors	≥75 kW Motors, MV & HV Induction Motors	≥11 kW Motors, MV & HV Induction Motors	All Motor Types
Bearing	75 %	95 %	41 %	41 %	42 %	21 %
Stator related	9 %	2 %	37 %	36 %	13 %	35 %
Rotor related	6 %	1 %	10 %	9 %	8 %	44 %
Others	10 %	2 %	12 %	14 %	38 %	–

This discrepancy between industrial relevance and academic focus informed the development of a balanced and comprehensive dataset that captures commonly occurring faults—including bearing, rotor, misalignment, and insulation-related faults—under realistic variable operating conditions. The dataset includes both AC motor and HVAC belt-driven system data and is intended to bridge this gap by providing researchers with high-quality data that reflects actual field failure distributions. This data article complements an original research paper on fault classification by providing detailed raw sensor measurements, annotated fault conditions, and multi-domain operational contexts.

## 3 Data Description

This article presents two case studies involving fault diagnosis of AC motors and rotating equipment under various mechanical and electrical conditions. Case study I is an AC motor testbed that simulates winding, bearing, misalignment, and journal bearing faults. Case Study II is an HVAC testbed that simulates belt loosening in a belt-connected HVAC system.

### 3.1 Case study I: AC motor testbed under constant or varying speed

In Case Study I, a comprehensive fault dataset was constructed using a testbed composed of AC motors with different capacities and fault types. This dataset includes vibration, current, and torque measurements under both constant and variable speed conditions.

In this case study, four motor fault types were simulated: misalignment, bearing fault, winding fault, and journal bearing clearance fault. The testbed includes AC motors with rated capacities of 1 HP, 3 HP, and 5 HP. Additionally, a 7.5 HP AC motor was modified by replacing its ball bearing with a journal bearing to simulate clearance-induced shaft vibration faults. The severity of each fault is expressed in Table 2.

**Fig. 1:**
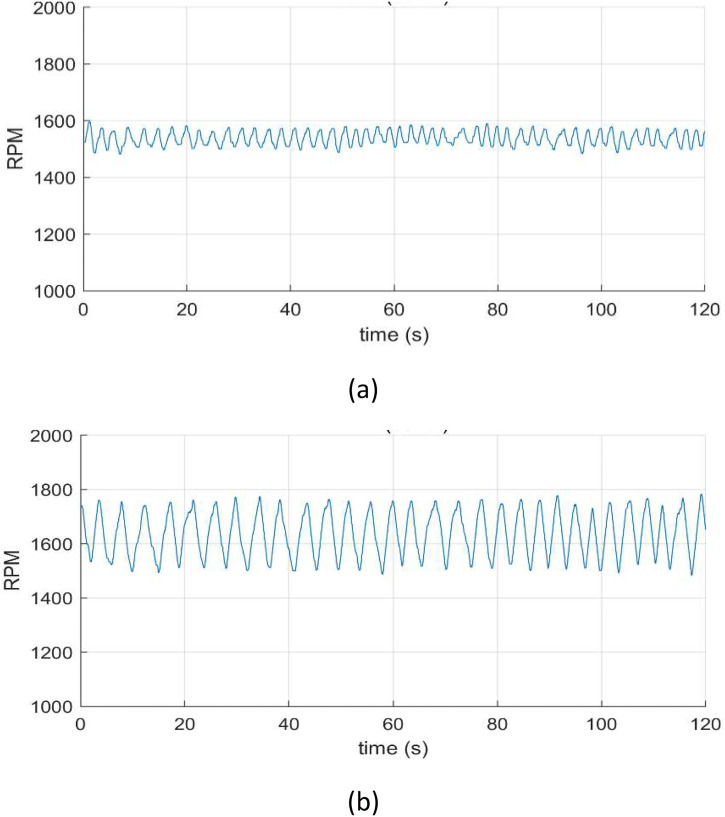
Rotating speed: (a) random speed fluctuations of 0 %∼4 % (50 Hz ∼ 52 Hz), and (b) random speed fluctuations of 0 %∼16 % (50 Hz ∼ 58 Hz).

Measurements were conducted under two speed conditions: constant speed and variable speed. To avoid interference from the 60 Hz power frequency, constant speed tests were performed by driving the motors at 50 Hz. Variable speed conditions were achieved by using a VFD (Variable Frequency Drive), with random speed fluctuations of 0 %∼4 % and 0 %∼16 % from the base speed to replicate realistic operating conditions as shown in Fig. 1. For each motor, loads of 0 %, 30 %, 60 %, and 90 % were applied based on the rated capacity using a hysteresis brake (AHB-10A, Magtrol Inc.), capable of up to 10 Nm load.

Vibration signals were acquired using accelerometers mounted on the vertical (z-axis) of the motor housing. For the constant speed tests, PCB352C34 sensors were used, while variable speed tests employed PCB356A45 sensors. Both sensors provide accurate measurements within the frequency range of 0.5–10 kHz with ±5 % tolerance, and a dynamic range up to 50 g pk.

Current signals were measured for all three phases (R, S, T) using Hioki CT7731 current sensors, which support both AC and DC current measurements up to 100 A. Torque signals were collected via a YDR 2 K torque meter (SETech Inc.) with a range of up to 19.6 Nm.

**Fig. 2:**
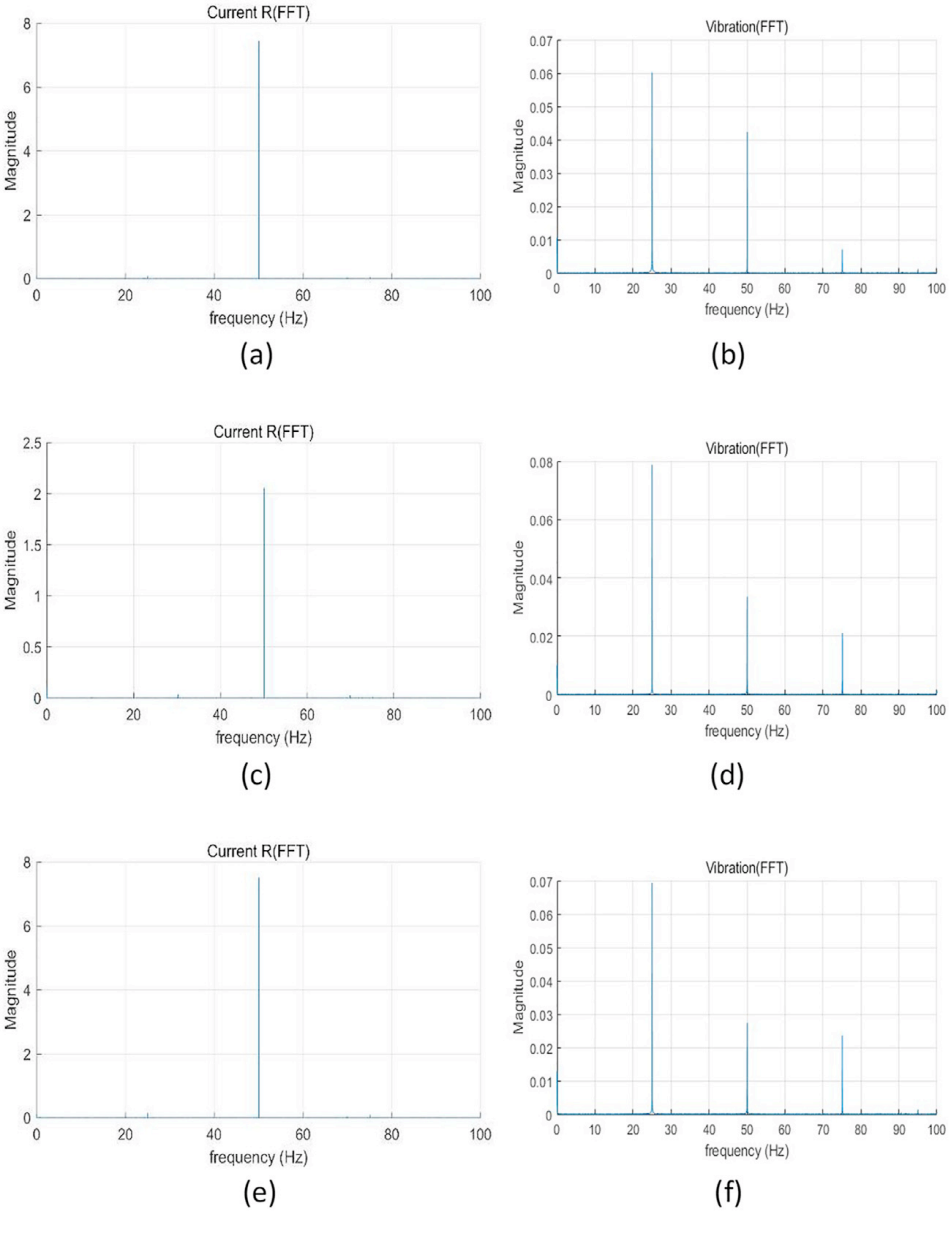

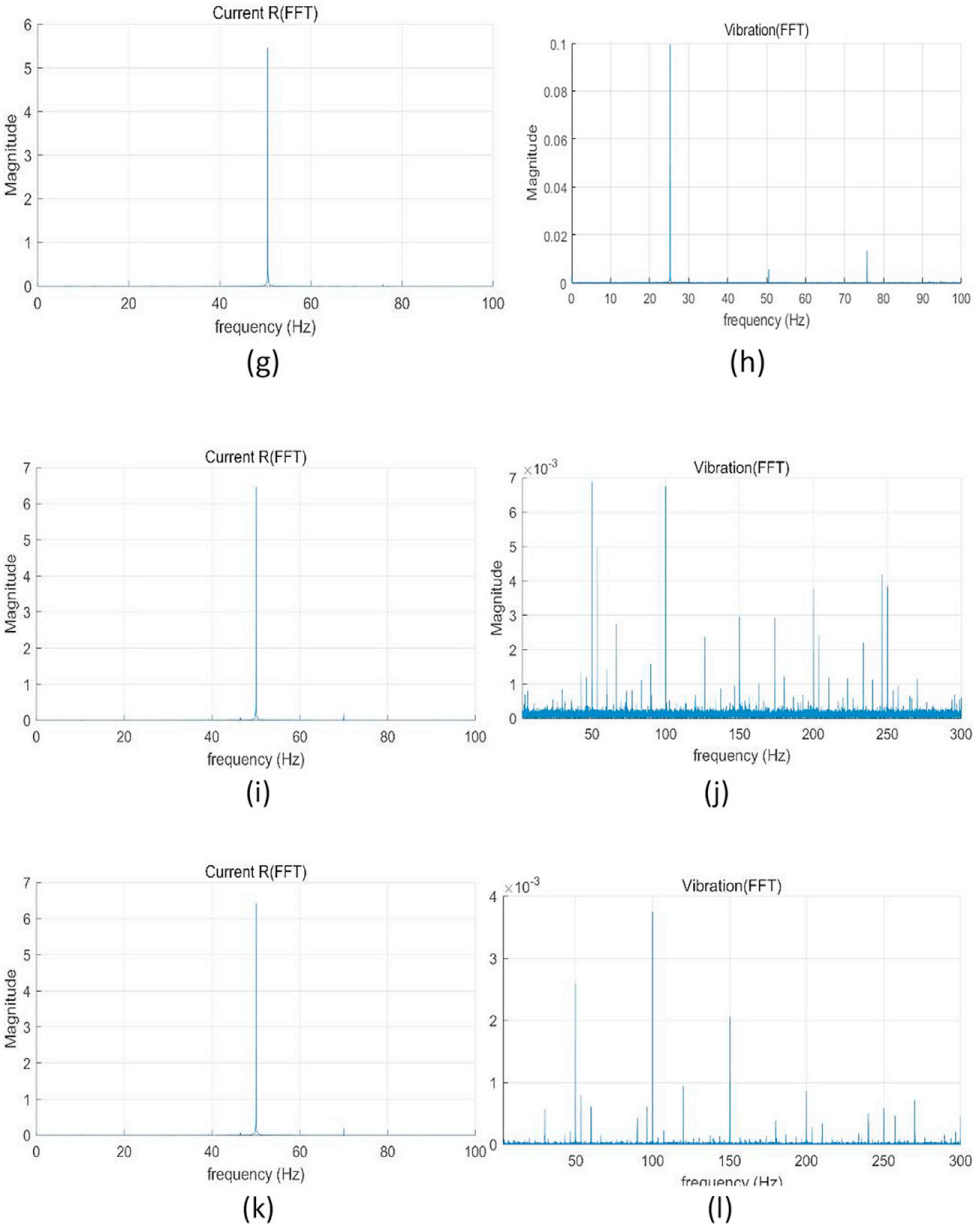
FFT results under constant speed: (a) current at normal, (b) vibration at normal, (c) current at winding fault (coil-coil; 60 %), (d) vibration at winding fault (coil-coil; 60 %), (e) current at misalignment 50 mm, (f) vibration at misalignment 50 mm, (g) current at bearing fault, (h) vibration at bearing fault, (i) current at journal bearing fault (0.75 µm), (j) vibration at journal bearing fault (0.75 µm), (k) current at journal bearing fault (0.95 µm), and (l) vibration at journal bearing fault (0.95 µm).

**Fig. 3:**
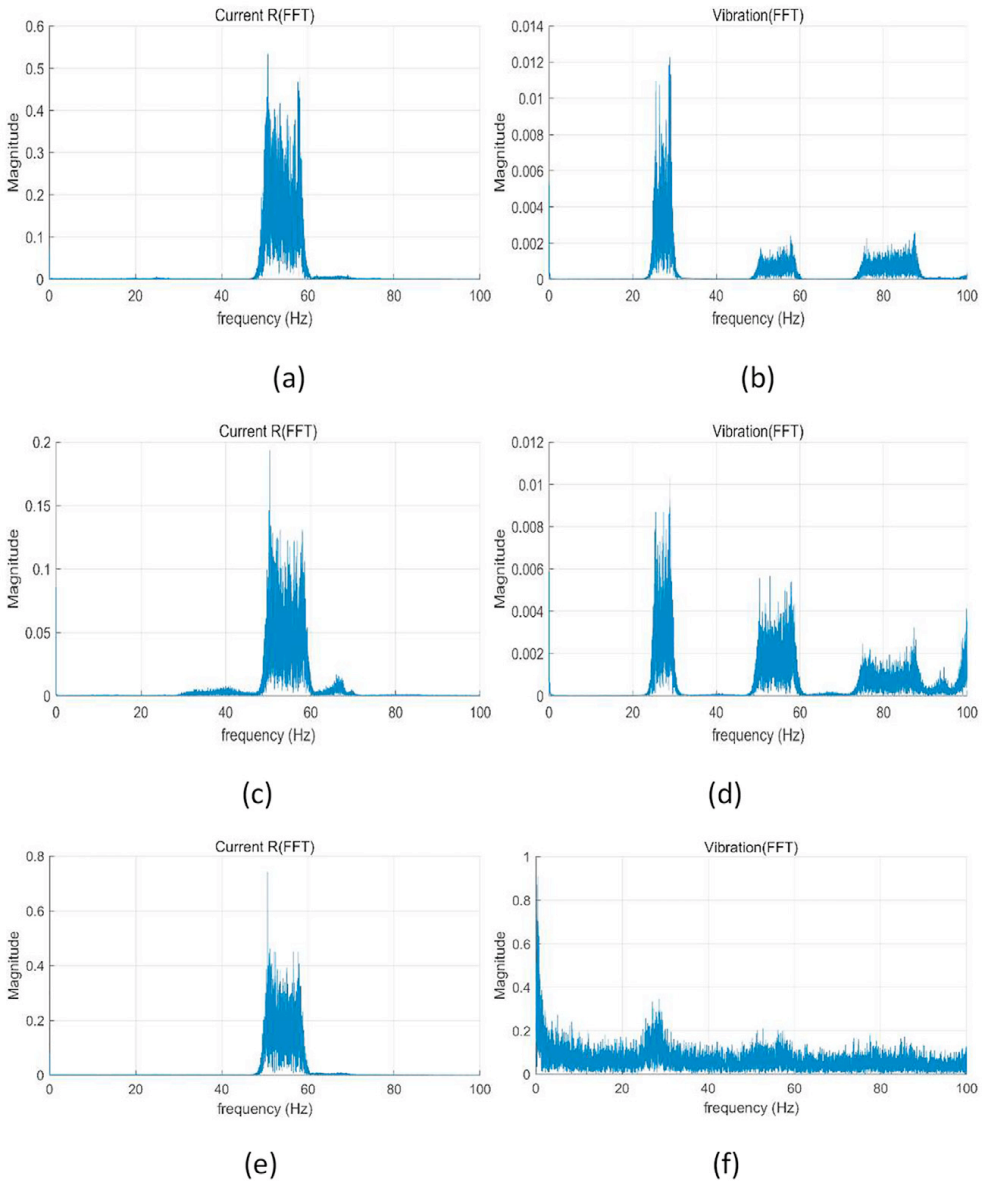

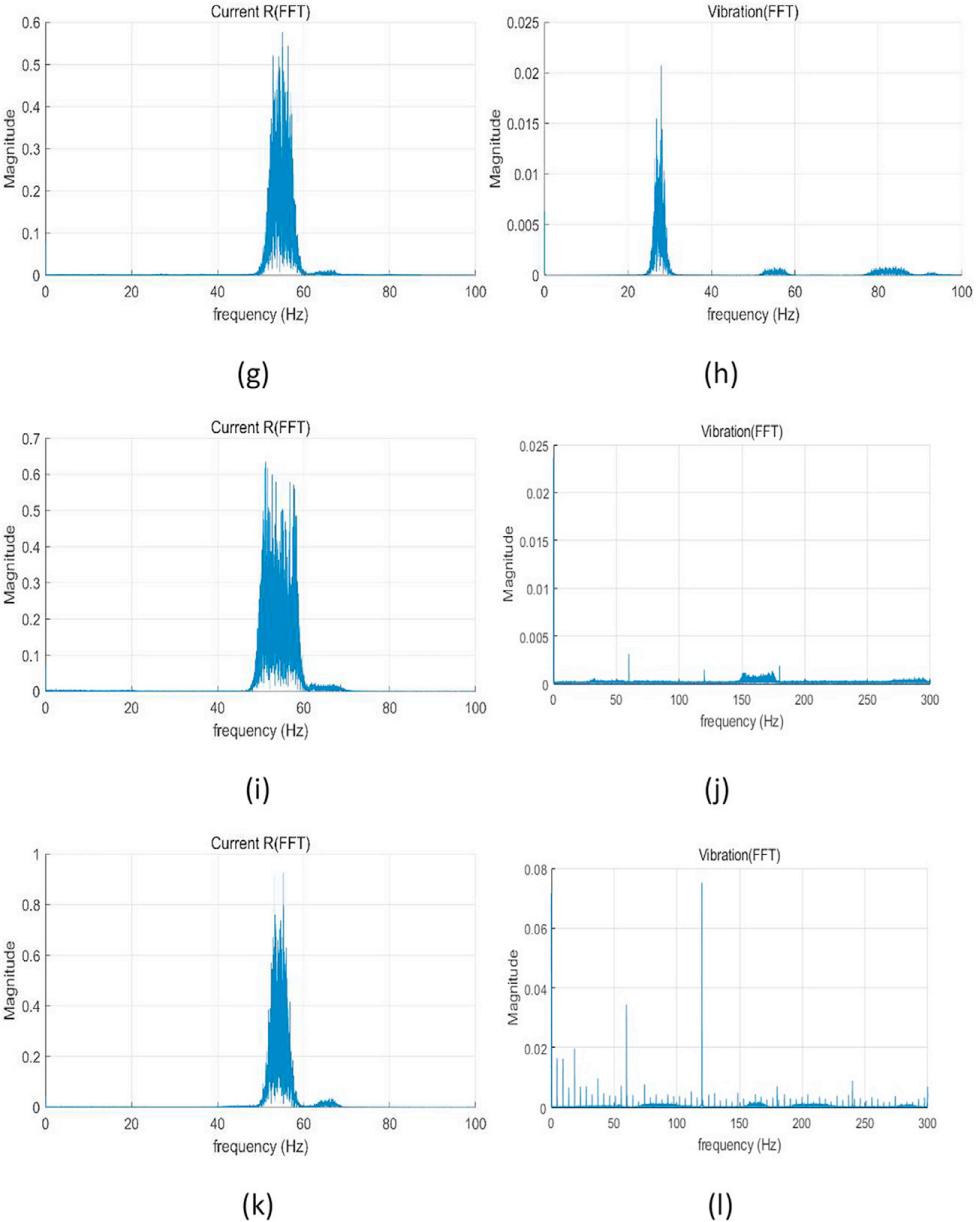
FFT results under random speed fluctuations of 0 %∼16 % (50 Hz∼58 Hz): (a) current at normal, (b) vibration at normal, (c) current at winding fault (coil-coil; 60 %), (d) vibration at winding fault (coil-coil; 60 %), (e) current at misalignment 50 mm, (f) vibration at misalignment 50 mm, (g) current at bearing fault, (h) vibration at bearing fault, (i) current at journal bearing fault (0. 75 µm), (j) vibration at journal bearing fault (0. 75 µm), (k) current at journal bearing fault (0. 95 µm), and (l) vibration at journal bearing fault (0. 95 µm).

All sensors were connected to a data acquisition system consisting of NI 9234 and NI 9215 modules, mounted on a cDAQ-9185 chassis. The sampling rates were set based on sensor and DAQ specifications: vibration and torque signals were sampled at 25.6 kHz, and current signals at 100 kHz. The recording duration was 300 s under constant speed conditions and 120 s under variable speed conditions. Sample data (vibration, and current) analysis using fast Fourier transform (FFT) are shown in Fig. 2, Fig. 3. The overall structure of the data is described in Table 3.

**Table 2:** Severity level of case study I.

Fault Types	Severity Level
Normal	–
Misalignment	10 mm, 30 mm, 50 mm
Bearing	Single spalling damage on ball surface
Winding (Coil-Coil)	10 %, 40 %, 60 %
Winding (Inter-Turn)	10 %, 40 %, 60 %
Journal Bearing	0.75 µm, 0.85 µm, 0.95 µm

**Table 3:** Description of dataset in case study I.

Data types	Speed condition	Motor Capacity	Fault types	Length (second)	Load (Nm)	Rotating speed (RPM)	Sampling rate (kHz)
Current, Vibration, Torque	Constant	1 HP	Normal, Misalignment, Bearing, Winding (Coil-Coil), Winding (Inter-Turn)*	300	0 %, 30 %, 60 %, 90 %	1450 (50 Hz)	Current: 100 Vibration: 25.6 Torque: 25.6
		3 HP		300	0 %, 30 %, 60 %	1466 (50 Hz)	
		5 HP		300	0 %, 30 %	1466 (50 Hz)	
		7.5 HP	Journal Bearing	300	0 %, 30 %, 60 %	2933 (50 Hz)	
	Random Varying (4 %)	1 HP	Normal, Misalignment, Bearing, Winding (Coil-Coil), Winding (Inter-Turn) *	120	0 %, 30 %, 60 %, 90 %	1450 ∼ 1508 (50 Hz ∼ 52 Hz)	
		3 HP		120	0 %, 30 %, 60 %	1466 ∼ 1525 (50 Hz ∼ 52 Hz)	
		5 HP		120	0 %, 30 %	1466 ∼ 1525 (50 Hz ∼ 52 Hz)	
		7.5 HP	Journal Bearing	120	0 %, 30 %, 60 %	2933 ∼ 3050 (50 Hz ∼ 52 Hz)	
	Random Varying (16 %)	1 HP	Normal, Misalignment, Bearing, Winding (Coil-Coil), Winding (Inter-Turn) *	120	0 %, 30 %, 60 %, 90 %	1450 ∼ 1682 (50 Hz ∼ 58 Hz)	
		3 HP		120	0 %, 30 %, 60 %	1466 ∼ 1701 (50 Hz ∼ 58 Hz)	
		5 HP		120	0 %, 30 %	1466 ∼ 1701 (50 Hz ∼ 58 Hz)	
		7.5 HP	Journal Bearing	120	0 %, 30 %, 60 %	2933 ∼ 3402 (50 Hz ∼ 58 Hz)	

⁎ Winding (Inter-Turn) faults are collected only under 0 % Load conditions.

Data files are provided in binary MATLAB (.mat) format. Each file contains synchronized time-series data for vibration (z-axis), torque measurements, and three-phase current (R-, S-, T-phase). The filenames indicate the fault type, motor capacity, load condition, and speed profile used in each test.

This dataset is compressed in parts due to data size issues. Therefore, you need to download a total of 6 split compressed files (“AC.z01” ∼ ”AC.z06”) and 1 main compressed file (“AC.zip”) in the same space and unzip "AC.zip". The download links for each are as follows.

• Title: AC Motor Testbed under constant or varying speed (“AC.z01” ∼ “AC.z06” and "AC.zip")
• Data identification number:
  10.17632/wg6h28cfbs.1
  10.17632/mvdg4t6cz7.1
  10.17632/697w94bhw2.1
  10.17632/j2r275rmdf.1
  10.17632/gr8c9ckhxh.1
  10.17632/j8c6mf2gn8.1
  10.17632/9r82jppsn7.1
• Direct URL to data:
  https://data.mendeley.com/datasets/wg6h28cfbs/1
  https://data.mendeley.com/datasets/mvdg4t6cz7/1
  https://data.mendeley.com/datasets/697w94bhw2/1
  https://data.mendeley.com/datasets/j2r275rmdf/1
  https://data.mendeley.com/datasets/gr8c9ckhxh/1
  https://data.mendeley.com/datasets/j8c6mf2gn8/1
  https://data.mendeley.com/datasets/9r82jppsn7/1

After unzip file, the sample structure of data is as follows:

1. Bearing_1HP_0 %.mat : This file includes the vibration (z-axis), torque measurements, and three-phase current (R-, S-, T-phase) in bearing fault of 1 HP motor under the 0 % load condition and constant speed.
2. Coil60 %_3HP_30 %.mat : This file includes the vibration (z-axis), torque measurements, and three-phase current (R-, S-, T-phase) in winding fault (60 % severity of coil-coil short) of 3 HP motor under the 30 % load condition and constant speed.
3. Bearing_1HP_0 %_52Hz.mat : This file includes the vibration (z-axis), torque measurements, and three-phase current (R-, S-, T-phase) in bearing fault of 1 HP motor under the 0 % load condition and speed variation (4 % random variation under constant speed conditions).
4. Bearing_1HP_0 %_58Hz.mat : This file includes the vibration (z-axis), torque measurements, and three-phase current (R-, S-, T-phase) in bearing fault of 1 HP motor under the 0 % load condition and speed variation (14 % random variation under constant speed conditions).

### 3.2 Case study II: belt looseness fault simulation in an HVAC testbed

Case Study II focuses on simulating and diagnosing belt looseness in a belt-driven HVAC (Heating, Ventilation, and Air Conditioning) system. The system is composed of a main motor connected to a fan via a belt and pulley mechanism. In this configuration, torque generated by the motor is transmitted to the fan through the belt. Any degradation in belt tension causes a mismatch between motor and fan torques, which results in measurable differences in vibration, current, and rotational speed signals.

The experiment was conducted under a constant speed operating condition by running the main motor at 60 Hz. The degree of belt looseness was controlled by shifting the motor away from the fan along a linear rail, thereby increasing the center-to-center distance between the pulleys. Five operating conditions were considered: one normal condition (denoted as d0), and four levels of looseness severity (d1 to d4). The severity adjustment and experimental layout are summarized in Table 4.

**Table 4:** Severity level of case study II.

Center distance	Condition
d0	Healthy belt (Normal)
d1	Weak belt looseness
d2	Moderate belt looseness
d3	Strong belt looseness
d4	Critical belt looseness

Vibration and current data were recorded continuously for 300 s per condition. Rotational speed (RPM) data were collected for a total of 500 s, saved as 500 separate “.txt” files, each representing a one-second window. Vibration and current data were stored in “.tdms” format, while RPM data were saved in ASCII text format.

Vibration signals (collected by PCB 352C34) were measured in the x- and y-directions (90° apart) at the main motor’s bearing housing in accordance with ISO standards. The order of the axes in the TDMS files is consistent with the x- and y-axes, respectively. Current signals (collected by Hioki CT7731) were captured from all three motor phases (R-, S-, T-phase), in that order. RPM (collected by Keyence FS-N41P) files contain three columns: time stamp, motor RPM, and fan RPM. Sampling frequencies were set according to the required precision for each sensor modality. Vibration was sampled at 25.6 kHz using NI 9234, and cDAQ-9185, while current and RPM signals were sampled at 100 kHz using NI 9215, and cDAQ-9185. The overall structure of the data is described in Table 5.

**Table 5:** Description of dataset in case study II.

Data types	Speed condition	Length (second)	Fault Types	Rotating speed (RPM)	Sampling rate (kHz)
Current	Constant	300	Normal (d0), d1, d2, d3, d4	1800 (60 Hz)	100
Vibration	Constant	300	Normal (d0), d1, d2, d3, d4	1800 (60 Hz)	25.6
RPM	Constant	500 (1 per file)	Normal (d0), d1, d2, d3, d4	1800 (60 Hz)	100

**Fig. 4:**
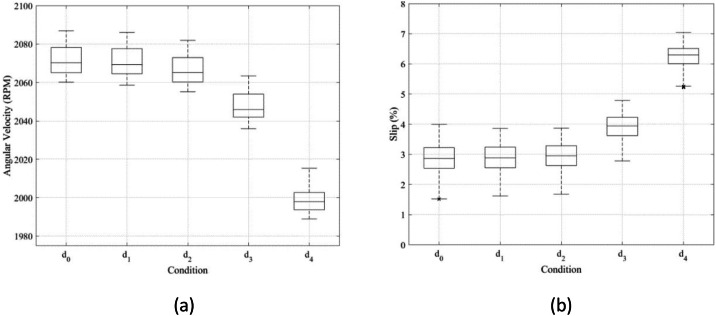
Simple data analysis: (a) Angular velocity of pulley at fan end according to belt looseness condition, and (b) Slip of belt-pulley system according to belt looseness condition.

To assess the impact of belt looseness, the angular velocity of the motor and the fan was analyzed [6]. As shown in Fig. 4(a), fan speed gradually decreases as looseness severity increases, indicating ineffective torque transmission. This behavior was quantified using a feature called slip, defined as the mismatch in angular speed between the motor and fan, normalized by the pulley diameters:

Slip is defined and calculated by:

(1) $$S=\left(1-\frac{D_{\text{fan}}}{D_{\text{motor}}}\times \frac{\Omega_{\text{fan}}}{\Omega_{\text{motor}}}\right)\times 100$$

where *S* is slip, $\Omega_{\text{motor}}$ is rotation speed of motor, $\Omega_{\text{fan}}$ is rotation speed of fan, $D_{\text{motor}}$ is diameter of pulley at motor, and $D_{\text{fan}}$ is diameter of pulley at fan. The diameter of each pulley is given as $D_{\text{motor}}$ =6.5 cm and *D*_fan_ =5.5 cm. In terms of comparing this dataset over fault severity, slips of each condition overlap each other, making it challenging to distinguish between them as shown in Fig. 4(b).

This dataset can be downloaded from the link below and consists of a single “HVAC.zip” file. Unzip the zip file to get the dataset.

• Title: Belt Looseness Fault Simulation in an HVAC Testbed
• Data identification number: 10.17632/5tt3zb2pns.1
• Direct URL to data: https://data.mendeley.com/datasets/5tt3zb2pns/1

The sample structure of data is as follows:

1. Current_D0.tdms : This file includes three-phase current (R-, S-, T-phase) in normal condition.
2. Vib_D0.tdms : This file includes vibration (x-, y-axis) in normal condition.
3. Current_D1.tdms : This file includes three-phase current (R-, S-, T-phase) in d1 center distance.
4. Vib_D1.tdms : This file includes vibration (x-, y-axis) in d1 center distance..
5. Current_D3.tdms : This file includes three-phase current (R-, S-, T-phase) in d3 center distance.
6. Vib_D3.tdms : This file includes vibration (x-, y-axis) in d3 center distance.
7. data_1.txt : This file includes RPM (motor, fan) at first of 1 s files.
8. data_100.txt : This file includes RPM (motor, fan) at 100th of 1 s files.

## 4 Experimental Design, Materials and Methods

### 4.1 Case study I: AC motor testbed under constant or varying speed

**Fig. 5:**
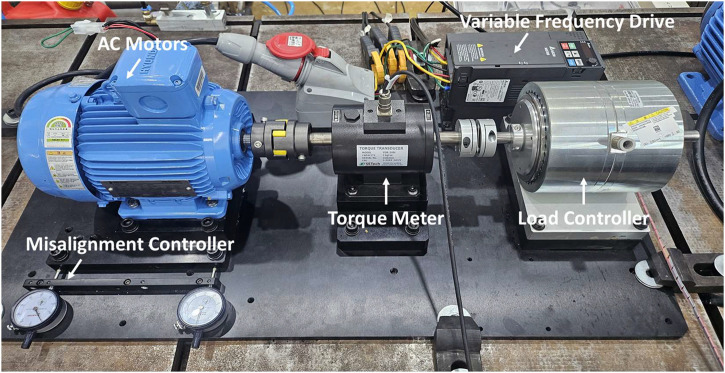
Description of AC motor testbed (Case study I).

The experimental data were acquired from an AC motor fault diagnosis testbed designed to simulate multiple mechanical and electrical faults under both constant speed and variable speed conditions. As shown in Fig. 5, the testbed consists of a main motor, torque transducer, and hysteresis brake. The motor is driven by either a fixed 50 Hz signal or a Variable Frequency Drive (VFD) with random speed variation. Torque is measured via the torque meter installed between the motor and the brake, and variable load is applied by controlling the braking torque.

Four types of AC motors were installed sequentially, with rated capacities of 1 HP, 3 HP, 5 HP, and 7.5 HP. The 7.5 HP motor was exclusively used for journal bearing fault simulation. Each motor was tested under different load levels, specifically 0 %, 30 %, 60 %, and 90 % of its rated torque capacity, as summarized in Table 6. In some cases, certain load conditions were omitted for safety reasons.

1. Misalignment Fault Simulation
  Misalignment was introduced by vertically displacing the shaft at the motor flange using a mechanical misalignment indicator. Only upward displacement was applied to simulate increasing severity levels of misalignment (10 mm, 30 mm, and 50 mm). Shaft misalignment introduces additional radial forces that result in measurable changes in vibration and torque signals.
2. Bearing Fault Simulation
  Bearing faults were induced in 1 HP, 3 HP, and 5 HP motors. As shown in Fig. 6, ball defects were fabricated using spark erosion to create spalls on the surface of the rolling elements. The focus was restricted to ball faults due to their distinct and frequent occurrence in bearing degradation processes, and their non-stationary signature behavior makes them suitable for advanced condition monitoring studies.

**Fig. 6:**
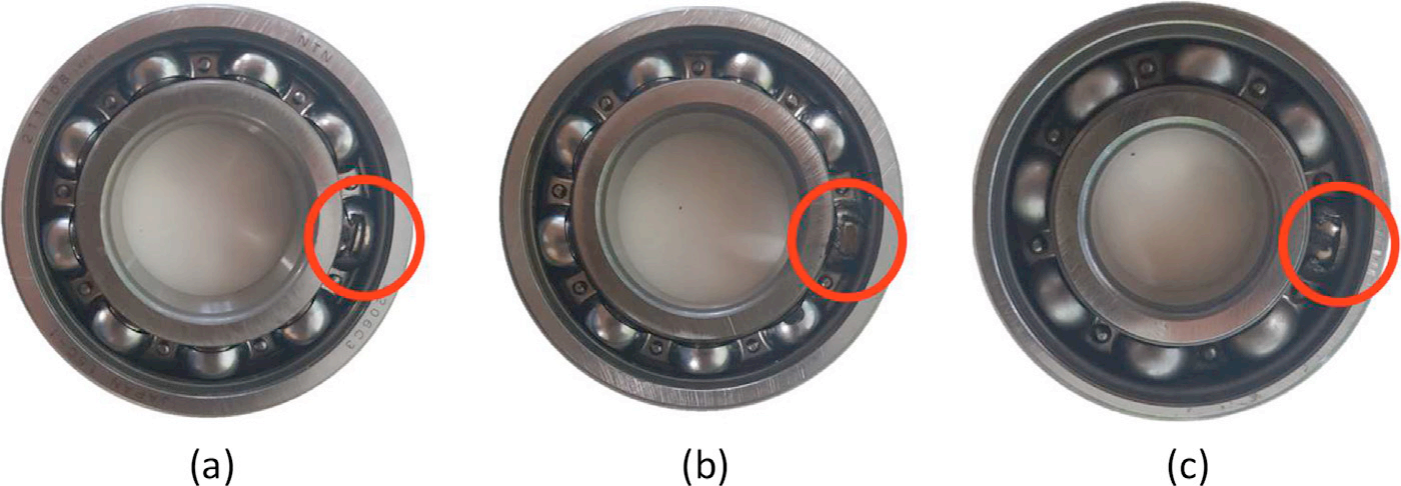
Ball bearing fault seeding in case study I: (a) 1 HP, (b) 3 HP, and (c) 5 HP.
3. Winding Fault Simulation
  Winding faults were introduced in the form of inter-turn short circuits. Variable resistors were connected to the stator windings, allowing controlled leakage current across specific coils. As shown in Fig. 7, this setup enabled simulation of three severity levels: 10 %, 40 %, and 60 % deviation from the nominal current flow. The winding faults were only applied to 1 HP, 3 HP, and 5 HP motors.

**Fig. 7:**
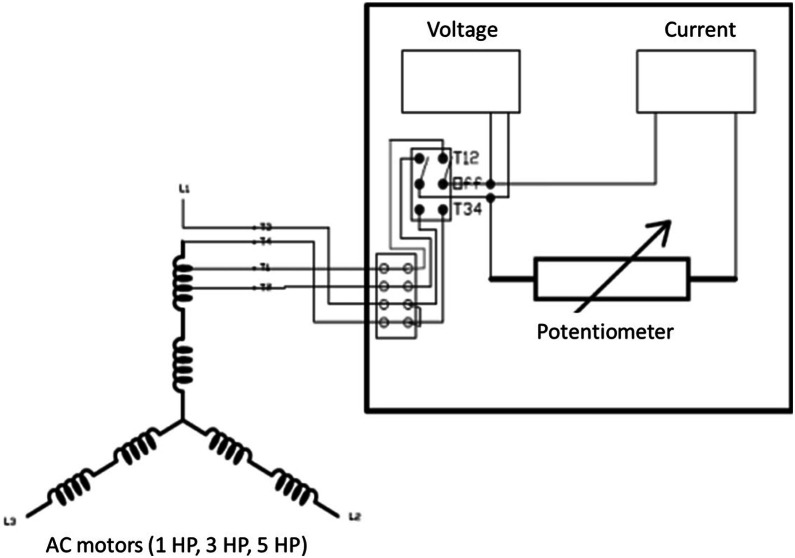
Winding fault seeding in case study I.
4. Journal Bearing Fault Simulation
  The 7.5 HP motor was structurally modified to include a journal bearing system in place of the original ball bearings. As shown in Fig. 8, an oil circulation system with a mechanical pump was constructed to provide lubrication. The original bearing housing was replaced with a machined journal bearing section (Fig. 8b), and mechanical sealing was employed to prevent oil leakage. Fault severity was defined based on the clearance between the shaft and bearing inner diameter (0.75 um, 0.85 um, and 0.95 um) (Fig. 8c). As the clearance increased, shaft instability and eccentricity became more pronounced, significantly influencing both vibration and current signals.

**Fig. 8:**
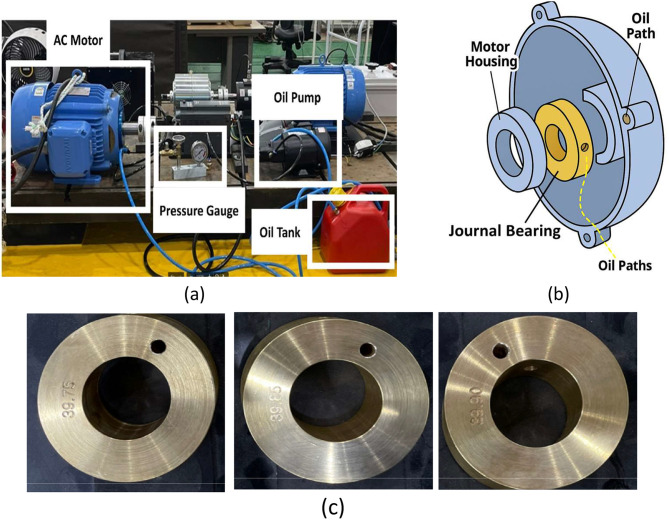
Journal bearing fault seeding in case study I: (a) Description of oil circulation, (b) Journal bearing insertion diagram, and (c) samples of journal bearing (0.75 µm, 0.85 µm, 0.95 µm).

**Table 6:** Specification of motor in case study I.

Motor Capacity	Company	Model Name	Max. Torque	Rated Voltage	Rated Frequency	Number of Phase / Number of Poles	Bearing
1 HP	Hyundai Electric	HS080AL202	3.92 Nm	380 V	60 Hz (1740 RPM)	4	6204ZZ
3 HP	Hyundai Electric	HS105AL202T	11.77 Nm	380 V	60 Hz (1760 RPM)	4	6206ZZC3
5HP	Hyundai Electric	HS113AL202T	19.62 Nm	380 V	60 Hz (1760 RPM)	4	6206ZZC3
7.5 HP	Hyundai Electric	HS131SR102	14.72 Nm	380 V	60 Hz (3520 RPM)	2	6208ZZC3

All data were recorded using National Instruments hardware (cDAQ-9185 chassis) with NI-9234 and NI-9215 modules. The vibration and torque signals were sampled at 25.6 kHz, and the three-phase current signals were sampled at 100 kHz. The total measurement duration was 300 s under constant speed conditions and 120 s under variable speed conditions.

### 4.2 Case study II: belt looseness fault simulation in an HVAC testbed

This case study investigates the belt looseness fault in a commercial air handling unit (AHU), which is a key component in heating, ventilation, and air conditioning (HVAC) systems. Belt slip, caused by insufficient tension between the motor and fan pulleys, is a crucial feature for fault detection. However, in practical applications, fluctuating rotation speeds of the motor and fan complicate slip monitoring. These fluctuations become more severe as belt looseness increases, making slip detection even more challenging. Since the primary role of the belt is to transfer torque, looseness directly affects the motor’s electrical load, which is reflected in the current signals.

**Fig. 9:**
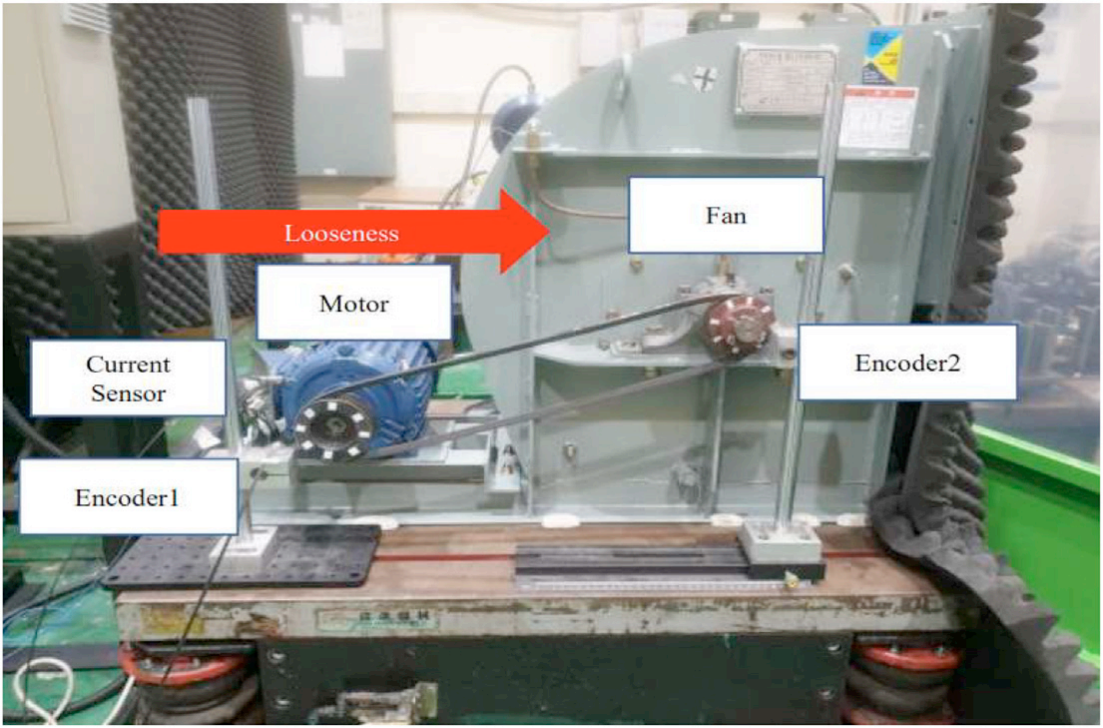
Description of HVAC testbed (Case study II).

To emulate these real-world conditions, a commercial HVAC unit was purchased and modified for experimental testing, as illustrated in Fig. 9. The system configuration consists of a main motor connected to a fan via a single belt. Belt looseness was induced by moving the motor along a linear track in the horizontal direction, increasing the center distance between the motor and fan pulleys.

The main motor used in this test was a 1.5 kW, 3-phase, 4-pole induction motor (Hyosung, South Korea), rated for 1750 RPM at 60 Hz. The system was operated at 380 V. While the motor was driven at 60 Hz, speed fluctuations were observed in both the motor and fan shafts, particularly under severe belt looseness conditions. These fluctuations correlate with increased slip, as the belt fails to maintain consistent torque transmission. To quantify the slip between the motor and fan, the rotational speeds of both shafts were measured using optical encoders. The slip was calculated using the following expression Eq. (1), where the motor pulley diameter is 6.5 cm and the fan pulley diameter is 5.5 cm.

Vibration sensors were mounted on the motor housing at the bearing position, aligned in x- and y-directions with 90° spacing, according to ISO standards. The system was evaluated under five conditions: one normal state (d0) and four belt looseness levels (d1 to d4), adjusted by incrementally displacing the motor to the right. Vibration and current data were collected for 300 s per condition and stored in single “.tdms” files. RPM data were collected for 500 s and stored in 500 separate “.txt” files, with one file per second. Each vibration file contains x-axis vibration, and y-axis vibration in two columns. Each current file contains R-phase, S-phase, and T-phase in three columns. Each RPM file contains time, motor RPM, and fan RPM in three columns.

## Limitations

While the dataset captures a wide variety of faults and conditions, it is limited to laboratory-scale testbeds and does not include long-term degradation or environmental noise seen in field deployments. Additionally, journal bearing faults were simulated mechanically and may not fully replicate real wear mechanisms.

## Ethics Statement

Human Lab., Center for Noise and Vibration Control Plus, Department of Mechanical Engineering, Korea Advanced Institute of Science and Technology, Daejeon, South Korea have read and follow the ethical requirements for publication in Data in Brief and confirming that the current work does not involve human subjects, animal experiments, or any data collected from social media platforms.

## CRediT authorship contribution statement

**Wonho Jung:** Conceptualization, Methodology, Software, Validation, Visualization, Writing – original draft, Writing – review & editing. **Junho Kim:** Data curation, Validation, Investigation. **Kangmin Jang:** Data curation, Validation. **Sung-Hyun Yun:** Data curation, Validation, Investigation. **Daeguen Lim:** Data curation, Validation, Investigation. **Minje Jin:** Investigation. **Yong-Hwa Park:** Funding acquisition, Supervision.

## Data Availability

Mendeley DataAC Motor Testbed under constant or varying speed (AC.z02) (Original data). Mendeley DataAC Motor Testbed under constant or varying speed (AC.z01) (Original data). Mendeley DataAC Motor Testbed under constant or varying speed (AC.zip) (Original data). Mendeley DataAC Motor Testbed under constant or varying speed (AC.z06) (Original data). Mendeley DataAC Motor Testbed under constant or varying speed (AC.z05) (Original data). Mendeley DataAC Motor Testbed under constant or varying speed (AC.z04) (Original data). Mendeley DataAC Motor Testbed under constant or varying speed (AC.z03) (Original data). Mendeley DataBelt Looseness Fault Simulation in an HVAC Testbed (Original data).
